# Mosquito immune responses and compatibility between Plasmodium parasites and anopheline mosquitoes

**DOI:** 10.1186/1471-2180-9-154

**Published:** 2009-07-30

**Authors:** Giovanna Jaramillo-Gutierrez, Janneth Rodrigues, Georges Ndikuyeze, Michael Povelones, Alvaro Molina-Cruz, Carolina Barillas-Mury

**Affiliations:** 1 Laboratory of Malaria and Vector Research, National Institute of Allergy and Infectious Diseases, National Institutes of Health, Rockville, MD 29892, USA; 2 Immunology and Infection, Division of Cell and Molecular Biology, Faculty of Natural Sciences, Imperial College London, London, UK

## Abstract

**Background:**

Functional screens based on dsRNA-mediated gene silencing identified several *Anopheles gambiae *genes that limit *Plasmodium berghei *infection. However, some of the genes identified in these screens have no effect on the human malaria parasite *Plasmodium falciparum*; raising the question of whether different mosquito effector genes mediate anti-parasitic responses to different *Plasmodium *species.

**Results:**

Four new *An. gambiae *(G3) genes were identified that, when silenced, have a different effect on *P. berghei *(Anka 2.34) and *P. falciparum *(3D7) infections. Orthologs of these genes, as well as *LRIM1 *and *CTL4*, were also silenced in *An. stephensi *(Nijmegen Sda500) females infected with *P. yoelii *(17XNL). For five of the six genes tested, silencing had the same effect on infection in the *P. falciparum-An. gambiae *and *P. yoelii-An. stephensi *parasite-vector combinations. Although silencing *LRIM1 *or *CTL4 *has no effect in *An. stephensi *females infected with *P. yoelii*, when *An. gambiae *is infected with the same parasite, silencing these genes has a dramatic effect. In *An. gambiae *(G3), TEP1, LRIM1 or LRIM2 silencing reverts lysis and melanization of *P. yoelii*, while *CTL4 *silencing enhances melanization.

**Conclusion:**

There is a broad spectrum of compatibility, the extent to which the mosquito immune system limits infection, between different *Plasmodium *strains and particular mosquito strains that is mediated by TEP1/LRIM1 activation. The interactions between highly compatible animal models of malaria, such as *P. yoelii *(17XNL)-*An. stephensi *(Nijmegen Sda500), is more similar to that of *P. falciparum *(3D7)-*An. gambiae *(G3).

## Background

Mosquitoes transmit many infectious diseases, including malaria, lymphatic filariasis, yellow fever, and dengue. Among these diseases, malaria is by far the most costly in terms of human health. It is endemic to more than 100 countries and causes 550 million cases per year, with the highest mortality in children from sub-Saharan Africa. Malaria transmission to humans requires a competent mosquito species, as *Plasmodium *parasites must undergo a complex developmental cycle and survive the defense responses of their insect host. In Africa, *Anopheles gambiae *is the major vector of *Plasmodium falciparum *infection, which causes the most aggressive form of human malaria.

The *Plasmodium berghei *(murine malaria) model is one of the most widely used experimental systems to study malaria transmission. Gene silencing by systemic injection of double-stranded RNA (dsRNA) has proven to be a very useful tool to carry out functional genomic screens aimed at identifying mosquito genes that mediate anti-parasitic responses. In general, *Anopheles gambiae *is considered to be susceptible to *P. berghei *infection, because a high prevalence of infection can be achieved and parasites are only rarely melanized; however, silencing of either thioester-containing protein 1 (*TEP1*) [1], leucine-rich repeat immune protein 1 (*LRIM1*) [2], or *LRIM2 *(also called *APL1*, [3]), enhances *P. berghei *infection by 4–5 fold; indicating that, when these effector molecules are present, about 80% of parasites are eliminated by a lytic mechanism[1]. It is well documented that *An. gambiae *mosquitoes have a different transcriptional response to infection with *P. berghei *and *P. falciparum *[4,5] and genes such as *LRIM1 *and C-type lectin 4 (*CTL4*) [2], which limit or enhance *P. berghei *infection, respectively, do not affect *P. falciparum *infection in *An. gambiae *[6]. This raises the possibility that some antiplasmodial genes identified using the *P. berghei *malaria model may not be relevant to human malaria transmission.

More than 400 species of anopheline mosquitoes have been identified, but only 40 of them are considered to be important disease vectors [7]. Different anopheline species and even particular strains of mosquitoes vary widely in their susceptibility to infection with a given *Plasmodium *parasite species. For example, twelve different strains of *Anopheles stephensi *have been shown to have very different susceptibility to *P. falciparum *(Welch strain) infection [8]. Furthermore, susceptibility had a strong genetic component, which allowed selection of a *An. stephensi *strain (Nijmegen Sda500) that is highly susceptible to *P. falciparum *infection [8]. A strain of *An. gambiae *(L35) was selected to be highly refractory to infection with *Plasmodium cynomolgy *(primate malaria). The L35 strain melanizes *P. cynomolgy*, as well as several other *Plasmodium *species such as *P. berghei *(murine malaria), *Plasmodium gallinaceum *(avian malaria), and other primate malaria parasites such as *Plasmodium gonderi, Plasmodium inui*, and *Plasmodium knowlesi*. Interestingly, *P. falciparum *strains from the New World are also melanized effectively, but not those of African origin, suggesting that there are genetic differences between *P. falciparum *strains that affect their ability to infect *An. gambiae *[9]. The African strains of *P. falciparum *tested appeared to be better adapted to their natural mosquito vector. However, great differences in the level of resistance to *P. falciparum *infection have been documented in families derived from individual *An. gambiae *females collected in the field [3,10], and a small region of chromosome 2L is a major determinant of genetic resistance to infection [3].

*Drosophila melanogaster *can support the development of *Plasmodium gallinaceum *oocysts when cultured ookinetes are injected into the hemocele [11]. This observation opened the possibility of using a genetic approach to screen for *Drosophila *genes that affect *Plasmodium P. gallinaceum *infection[12]. Furthermore, silencing of orthologs (or family members) of five of these candidate genes in *An. gambiae *(G3 strain) demonstrated that four of them also affected *P. berghei *infection in the mosquito [12].

In this study we compare how silencing a set of genes identified in the *Drosophila *screen affects *Plasmodium *infection in different vector-parasite combinations. We conclude that there is a broad range of compatibility between different *Plasmodium *strains and particular mosquito strains that is determined by the interaction between the parasite and the mosquito's immune system. We define compatibility as the extent to which the immune system of the mosquito is actively limiting *Plasmodium *infection. For example, the *P. yoelii-An. stephensi *and *P. falciparum-An. gambiae *strains used in this study are highly compatible vector-parasite combinations, as silencing several genes involved in oxidative response or immunity has no significant effect on infection. In contrast, silencing the same genes has a strong effect in less compatible vector-parasite combinations such as *P. yoelii-An. gambiae *or *P. berghei-An. gambiae*.

## Results and discussion

### Effect of GSTT1 and GSTT2 silencing on *P. berghei *infection

**Figure 1:**
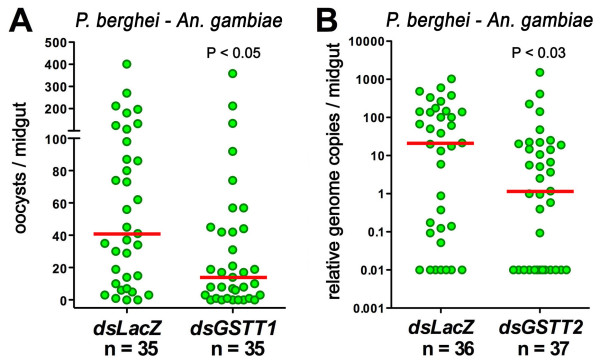
**Effect of silencing *An. gambiae *(G3) *GSTT1 *and *GSTT2 *on *P. berghei *infection**. Panel A, Effect of silencing glutathione-S-transferase theta-1 (*GSTT1*) on Plasmodium infection. GFP-expressing parasites were counted directly 6 days post infection (PI). Panel B, Effect of silencing glutathione-S-transferase theta-2 (*GSTT2*) on Plasmodium infection. Infection levels were determined based on the relative abundance of *P. berghei *28S and *An. gambiae *S7 genes in genomic DNA isolated from midguts 6 days PI. The dots represent the infection level on individual midguts, and the median infection level is indicated by the horizontal line. Distributions are shown using a logarithmic scale for *GSTT2 *and are compared using the Kolmogorov-Smirnov (KS) test; n = number of mosquitoes; P values lower than 0.05 are considered to be significantly different.

The effect of silencing *An. gambiae *orthologs (or homologs) of genes originally identified in the *Drosophila *genetic screen on *P. berghei *infectivity is summarized in Table 1[12]. Knockdown of arginine kinase (*ArgK*) and oxidation resistance gene 1 (*OXR1*) reduces infection. Tetraspanin and heat-shock cognate 3 (*Hsc-3*) silencing have the opposite effect, enhancing infection, while reducing the expression of the solute transporter (Sol. Trsp.) gene did not affect infection with *P. berghei *[12]. The effect of silencing two *An. gambiae *homologs of a glutathione S-transferase of the theta class (*GSTT*) (CG1702-PA) gene also identified in the *Drosophila *screen on *P. berghei *infection was evaluated. Injection of *GSTT1 *(AGAP000761-PA) or *GSTT2 *(AGAP000888-PA) dsRNA reduced mRNA expression by 60% and 55%, respectively, relative to the control groups injected with dsLacZ. Both *GSTT1 *and *GSTT2 *knockdown significantly reduce *P. berghei *infection (P < 0.05 and P < 0.03, respectively) using the Kolmogorov-Smirnov (KS) test (Figure 1 and Table 1).

**Table 1:** Effect of silencing seven *An. gambiae *genes or their orthologs in *An. stephensi *on the intensity of *P. berghei*, *P. falciparum *or *P. yoelii *infection.

*An. gambiae *Gene ID	Gene	***An. gambiae P. berghei ***(21°C)	***An. gambiae P. falciparum ***(26°C)	***An. stephensi P. yoelii ***(24°C)
**AGAP005627**	*ArgK*	*Decrease*^1^	*Decrease*	
**AGAP010892**	*Sol. trsp*.	No effect^1^	No effect	
**AGAP005233**	*Tetrasp*.	**Increase**^1^	**Increase**	

**AGAP001751**	*OXR1*	*Decrease*^1^	No effect	No effect
**AGAP004192**	*Hsc-3*	**Increase**^1^	*Decrease*	**Increase**
**AGAP000761**	*GSTT1*	*Decrease*	No effect	No effect
**AGAP000888**	*GSTT2*	*Decrease*	**Increase**	**Increase**
**AGAP006348**	*LRIM1*	**Increase**^2^	No effect.^3^	No effect
**AGAP005335**	*CTL4*	*Decrease*^2^	No effect.^3^	No effect

^1^Brandt *et al*., 2008

^2^Osta *et al*., 2004

^3^Cohuet *et al*., 2006

### Direct comparison of the effect of silencing seven *An. gambiae *genes on *P. berghei *and *P. falciparum *infection

**Figure 2:**
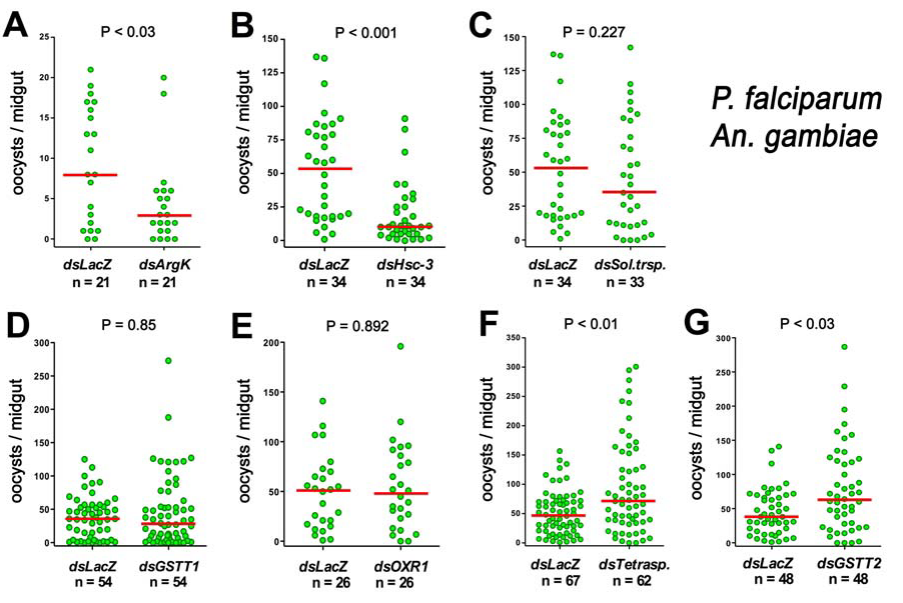
**Effect of silencing several *An. gambiae *(G3) genes on parasite *P. falciparum *infection**. Effect of silencing arginine kinase (*ArgK*) (Panel A), heat shock cognate 3 (*Hsc-3*) (Panel B), solute transporter (*Sol. Trsp.) *(Panel C), glutathione-S-transferase theta-1 (*GSTT1*) (Panel D), oxidation resistance gene 1 (*OXR1*) (Panel E) tetraspanin (*Tetrasp*.) (Panel F), and glutathione-S-transferase theta-2 (*GSTT2*) (Panel G) on *P. falciparum *infection. The number of *P. falciparum *oocysts present was determined by directly counting mercurochrome-stained parasites 7–8 days post infection. The dots represent the number of parasites present on individual midguts, and the median number of oocysts is indicated by the horizontal line. Distributions are compared using the Kolmogorov-Smirnov test; n = number of mosquitoes; P values lower than 0.05 are considered to be significantly different.

The effect of reducing expression of the five genes previously reported [12] as well as *GSTT1 *and *GSTT2 *in *An. gambiae *infected with *P. falciparum *(3D7 strain) was evaluated (Figure 2). Silencing of ArgK and Hsc-3 significantly reduced infection (P < 0.05 and P < 0.001, respectively, using the KS test) (Figure 2A, B). *Sol. Trsp*., *GSTT1*, and *OXR1 *silencing did not affect *P. falciparum *infection (Figure 2C–E), while tetraspanin and *GSTT2 *knockdown enhanced infection (P < 0.01 and P < 0.03; KS test) (Figure 2F, G). A summary of these results is shown in Table 1.

Silencing ArgK, *Sol. Trsp*., and tetraspanin genes has a similar effect on *P. berghei *and *P. falciparum *infection. ArgK is a key enzyme in cellular energy homeostasis in arthropods, with a function similar to that of creatine kinase in mammals. This enzyme catalyzes the synthesis of phosphoarginine, which serves as an energy reserve. The high-energy phosphate in phosphoarginine can be transferred to ADP to renew ATP during periods of high energy demand [13]. Apparently, silencing this enzyme results in a physiologic state in the mosquito that does not foster the development of either *P. berghei *or *P. falciparum*. Silencing of the solute transporter has no effect, while knockdown of tetraspanin enhances infection with both parasites. Tetraspanins are proteins with four transmembrane (TM) domains that are associated extensively with one another and with other membrane proteins to form specific microdomains distinct from lipid rafts. They are expressed on the surface of numerous cell types and are involved in diverse processes from cell adhesion to signal transduction and some of them inhibit the function of other members of the same family of proteins [14]. CD81 is a tetraspanin that has been shown to be required for hepatocyte invasion by *P. falciparum *and *P. yoelii *sporozoites [15]. Silencing of the *An. gambiae *tetraspanin gene may enhance parasite invasion and/or prevent the activation of an immune cascade that limits infection with *P. berghei *and *P. falciparum*.

*OXR1*, *GSTT1*, *GSTT2 *and *Hsc-3 *silencing has a different effect on *P. berghei *and *P. falciparum *infection. In yeast and mammals, *OXR1 *is induced by heat and oxidative stress and prevents oxidative damage by an unknown mechanism [16]. In *An. gambiae*, *OXR1 *silencing decreases resistance to oxidative challenge and prevents the induction of genes involved in ROS detoxification, such as catalase, following a blood meal (G. Jaramillo-Gutierrez and C. Barillas-Mury, unpublished). We have previously shown that higher ROS levels in *An. gambiae *reduce *P. berghei *infection [17]. Thus, it is likely that the decrease in *P. berghei *infectivity following *OXR1 *silencing is due to an increase in ROS. The unexpected observation that *OXR1 *silencing does not affect *P. falciparum *infection suggests that either this parasite species is less susceptible to oxidative stress or that the ingestion of human blood results in less accumulation of ROS in the mosquito.

GSTs play an important role as antioxidants and are involved in the detoxification of xenobiotics. GSTs of the epsilon and delta class have been extensively studied for their role in insecticide resistance in mosquitoes [18]. The GST-Theta1 (*GSTT1*) null genotype in human males is highly associated to increased risk of basal cell carcinoma of the skin [19]. Furthermore, in diabetics, the deletion of one copy of the *GSTT1 *gene is associated with elevated markers of inflammation and lipid peroxidation [20]. Therefore, silencing of *GSTT1 *and *GSTT2 *could result in increased lipid peroxidation, which is expected to be deleterious to *P. berghei*; however, it is not clear why reducing *GSTT2 *expression enhances *P. falciparum *infection.

### Susceptibility of *An. stephensi *(Nijmegen Sda500 strain) and *An. gambiae *(G3) to *P. yoelii *infection

The observed differences in the effect of silencing specific *An. gambiae *(G3 strain) genes on *P. berghei *and *P. falciparum *infection may reflect the degree of compatibility between these two parasite species and the mosquito strain used. Alternatively, mosquitoes may trigger different sets of effector genes in response to different *Plasmodium *species. To explore these possibilities, we evaluated the responses of two mosquito species that differ in their susceptibility to the same *Plasmodium *parasite.

**Figure 3:**
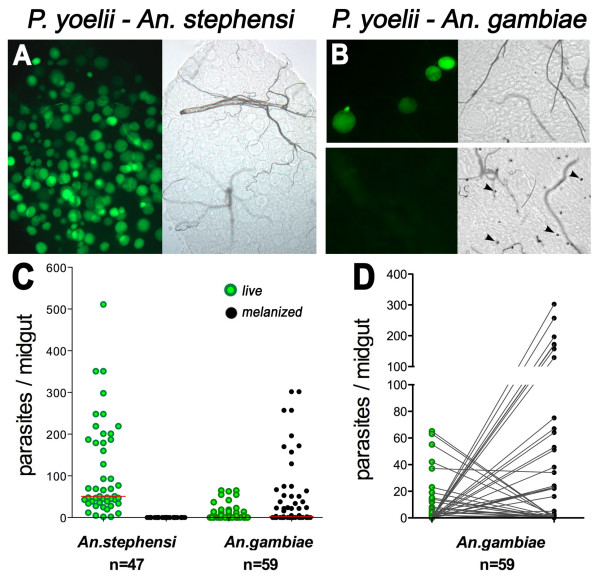
**Susceptibility of *An. stephensi *(Nijmegen Sda500) and *An. gambiae *(G3) to *P. yoelii *infection**. *An. stephensi *and *An. gambiae *mosquitoes were fed on the same *P. yoelii*-infected mouse. The images illustrate the level of infection and parasite melanization observed 6 days post infection (PI) in *An. stephensi *(Panel A) or *An. gambiae *(Panel B) females infected with *P. yoelii*. Live parasites are detected with green fluorescence (left panels), and those melanized are in DIC images (right panels). Panel C, Number of live (green dots) or melanized (black dots) parasites present on individual midguts 6 days PI. The median number of oocysts is indicated by the horizontal line. Distributions are compared using the Kolmogorov-Smirnov test; n = number of mosquitoes; P values lower than 0.05 are consider to be significantly different. Panel D, The number of live (green dots) and melanized (black dots) *P. yoelii *parasites on individual *An. gambiae *midguts is shown connected by a line. In most mosquitoes, either all parasites are alive or all are melanized. There are very few midguts in which both live and melanized parasites are observed.

The susceptibility of *An. stephensi *(Nijmegen Sda500), a strain highly susceptible to *P. falciparum *infection [8], and *An. gambiae *(G3) females to *P. yoelii *infection was compared by feeding them on the same infected mouse. *An. stephensi *is highly susceptible to *P. yoelii *infection, as no melanized parasites are observed and the median number of live oocysts is 51-fold higher than in *An. gambiae *(Figure 3A, C and Table 2). In contrast, *An. gambiae *(G3) is partially refractory and has two distinct phenotypes (Figure 3B). In approximately half of the mosquitoes, all parasites are melanized, while in the other half, parasite lysis appears to be the main defense response, as no melanizations are observed (Figure 3C, D). Interestingly, the prevalence of mixed phenotypes–that is, mosquitoes in which both live and melanized parasites are observed–is low (10%; Table 2). These results are in agreement with a previous report in which susceptibility of *An. gambiae *(G3) and *An. stephensi *(Pakistan) to *P. yoelii *infection was compared [21].

**Table 2:** *An. gambiae *(G3) and *An. stephensi *(Nijmegen Sda500) infections with *P. yoelii*.

Mosquito species	Prevalence of infection	Median live oocyst number	Oocyst range	% of midguts with melanized parasites	% of midguts with live and melanized parasites
***An. gambiae*** n = 59	52%	1	0–65	59%	10%
***An. stephensi*** n = 47	100%	51	2–302	0%	0%

### Effect of silencing *An. stephensi *orthologs on *P. yoelii *infection

**Figure 4:**
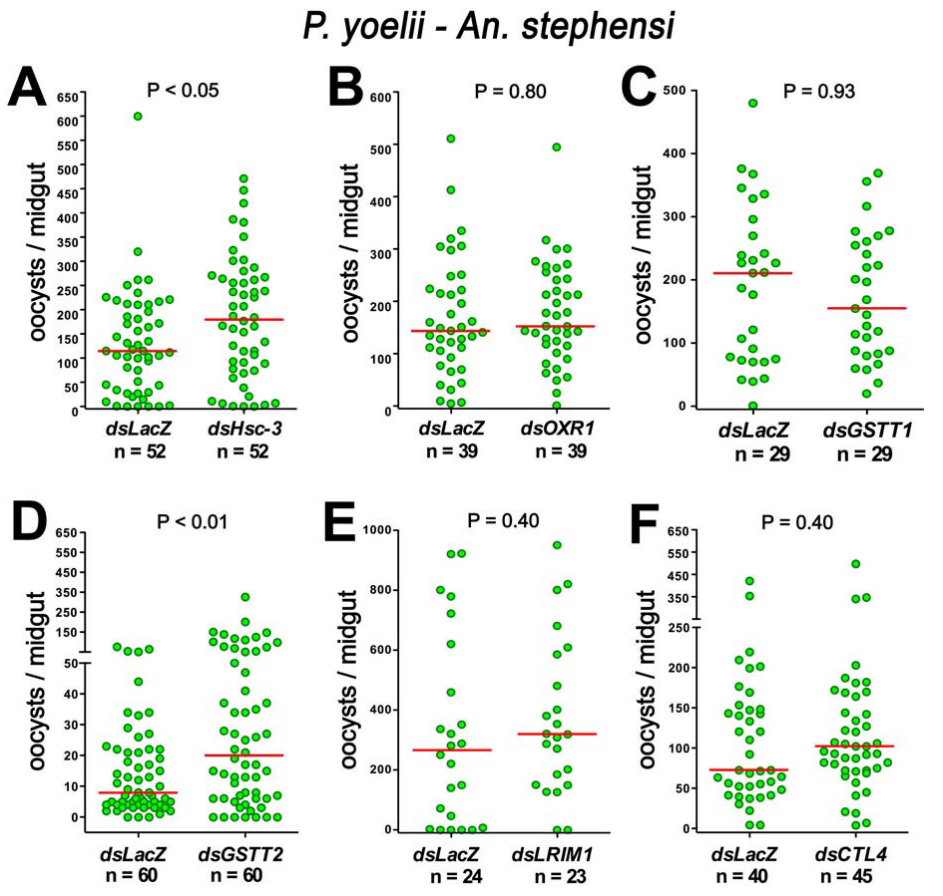
**Effect of silencing several *An. stephensi *(Nijmegen Sda500) genes on *P. yoelii *infection**. Effect of silencing heat shock cognate 3 (*Hsc-3*) (Panel A), oxidation resistance gene (*OXR1*) (Panel B), glutathione-S-transferase theta-1 (*GSTT1*) (Panel C), glutathione-S-transferase theta-2 (*GSTT2*) (Panel D), leucine rich-repeat immune protein 1 (*LRIM1*) (Panel E), and C-type lectin 4 (*CTL4*) (Panel F) on *P. yoelii *infection. The dots represent the number of oocysts present on individual midguts 6 days post infection. The median number of oocysts is indicated by the horizontal line. Distributions are compared using the Kolmogorov-Smirnov test; n = number of mosquitoes; P values lower than 0.05 are consider to be significantly different.

Six genes whose phenotypes differ when *An. gambiae *is infected with *P. berghei *or *P. falciparum *were examined. *An. stephensi *orthologs of *OXR1*, *Hsc-3*, *GSTT1*, and *GSTT2*, as well as two other genes previously reported in the literature (*LRIM1 *and *CTL4*), were silenced, and the effect on *P. yoelii *infection was evaluated. Five of the six genes tested had similar effects in the *An. gambiae*-*P. falciparum *and the *An. stephensi*-*P. yoelii *systems (Table 1). Silencing *OXR1*, *LRIM1*, *CTL4*, or *GSTT1 *had no effect, while *GSTT2 *and *Hsc-3 *silencing enhanced *P. yoelii *infection in *An. stephensi *(Figure 4 and Table 1). *Hsc-3 *was the only gene that gave a different phenotype between *An. gambiae*-*P. falciparum *and *An. stephensi*-*P. yoelii*. Conversely, this was also the only gene that had a similar phenotype in *An. gambiae *infected with *P. berghei *and in *P. yoelii*-infected *An. stephensi*. The expression of heat shock proteins is temperature dependent; thus the differences in the effect of *Hsc-3 *silencing in mosquitoes infected with different *Plasmodium *species could be due to physiologic differences resulting from the temperature at which infected mosquitoes are kept. For example, *Hsc-3 *silencing decreases *P. falciparum *infection (26°C) in *An. gambiae *but results in a significant but mild increase in *P. yoelii *infection (24°C) in *An. stephensi *and a strong enhancement of *P. berghei *infection (21°C) in *An. gambiae*. Interestingly, a decrease in parasite number is also observed in the *Drosophila *line in which a P-element has been inserted close to the *Hsc-3 *gene. In the fly system, *in vitro *cultured *P. gallinaceum *ookinetes are injected into the hemocele and the infected flies kept at 27°C [12]. It appears that silencing *Hsc-3 *decreases *Plasmodium *infection when the infected insects are kept at a higher temperature but has the opposite effect, enhancing infection, when infected insects are kept at a lower temperature.

### Refractoriness of *An. gambiae *(G3) to *P. yoelii *infection is due to activation of the mosquito immune system

**Figure 5:**
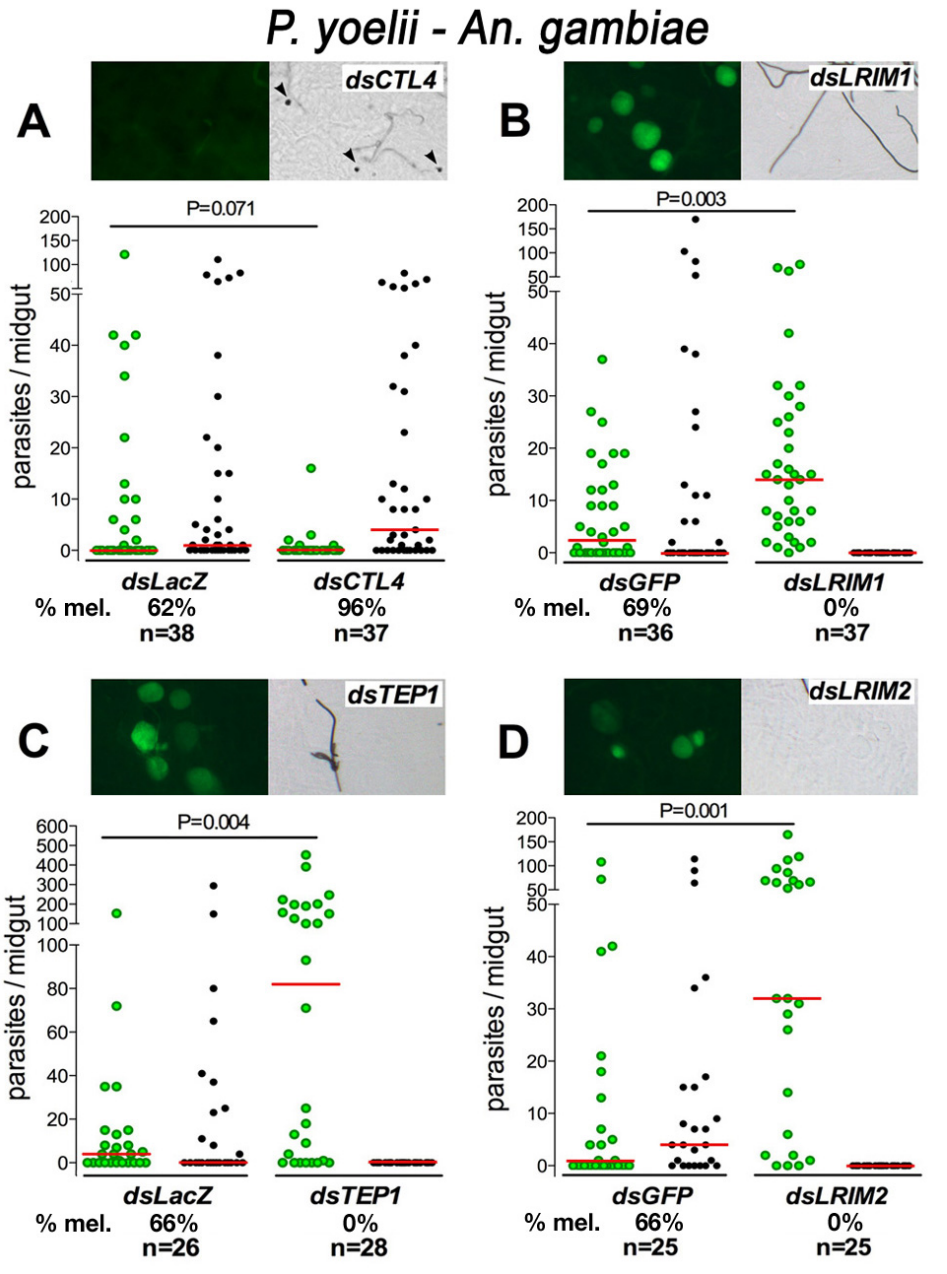
**Effect of silencing *An. gambiae *(G3) *CTL4, LRIM1, TEP1, or LRIM2 *on *P. yoelii *infection**. The images illustrate the level of infection and parasite melanization observed 6 days post infection (PI) when each gene was silenced. Live parasites are detected with green fluorescence (left panels) and those melanized are in DIC images (right panels). Effect of silencing C-type lectin 4 (*CTL4*) (Panel A), leucine rich-repeat immune protein 1 (*LRIM1*) (Panel B), thioester-containing protein 1 (*TEP1*) (Panel C), or leucine rich-repeat immune protein 2 (*LRIM2*) (Panel D) on *P. yoelii *infection. The dots represent the number of live (green) or melanized (black) parasites on individual midguts 6 days PI. The median number of oocysts is indicated by the horizontal line. Distributions are compared using the Kolmogorov-Smirnov test; n = number of mosquitoes; P values lower than 0.05 are consider to be significantly different.

The fact that *LRIM1 *and *CTL4 *silencing in *An. stephensi *(Nijmegen Sda500 strain) had no effect on *P. yoelii *infection could reflect a lack of activation of the immune system in this highly susceptible mosquito strain. Alternatively, it is also possible that *LRIM1 *and *CTL4 *do not participate in mosquito antiparasitic responses to *P. yoelii*. To explore these two possibilities, the effect of *CTL4 *and *LRIM1 *silencing in *An. gambiae *(G3) females, which are partially refractory to *P. yoelii *infection, was investigated. *CTL4 *silencing increases the number of melanized parasites from 62% to 95% (Figure 3A). Conversely, *LRIM1 *silencing completely reverts *P. yoelii *melanization and increases the median number of live oocysts by 4.6 fold (Figure 5B). To further investigate the participation of the *An. gambiae *immune system on the partial refractoriness of this species to *P. yoelii *infection, the effect of silencing *TEP1 *and *LRIM2 *was also evaluated. *TEP1 *and *LRIM2 *had a similar effect as *LRIM1*, enhancing infection by 32 and 20.5 fold, respectively (Figure 5C, D).

## Conclusion

The effect of silencing multiple mosquito genes in the highly compatible *P. yoelii *(17XNL)-*An. stephensi *(Nijmegen Sda500)system was very similar to that observed when *P. falciparum *(3D7) was used to infect *An. gambiae *(G3), its natural vector; suggesting that *P. yoelii-An. stephensi *is a representative animal model to study *P. falciparum *interactions with compatible vectors. Furthermore, *P. yoelii*-infected females can be kept at 24°C, a temperature that is more physiological for mosquitoes and closer to that used for *P. falciparum *infections (26°C).

Using less compatible parasite-mosquito combinations, such as the *P. berghei-An. gambiae *or *P. yoelii-An. gambiae *strains described in this study, may be particularly useful to identify and characterize immune pathways in the mosquito that could potentially limit human malaria transmission. Once a potential pathway is defined, it is possible to investigate if certain parasite strains avoid activating them, or if the effector genes are inefficient. It may also be possible to use alternative strategies (such as chemicals or fungal infections) to activate these potential antiplasmodial responses and test their effectiveness in limiting malaria transmission in natural vector-parasite combinations.

There is a broad spectrum of compatibility between different strains of *Plasmodium *and particular mosquito strains; for example, *An. gambiae *(G3) is highly compatible with *P. falciparum *(3D7) parasites, but has low compatibility with *P. yoelii *17XNL. A given strain of *Plasmodium *can also be more compatible with certain mosquitoes. For example, *P. yoelii *17XNL is much more compatible with *An. stephensi *(Nijmegen Sda500 strain) than with *An. gambiae *(G3). *TEP1 *silencing in *An. gambiae *(Keele strain) mosquitoes enhances infection with *P. falciparum *(NK54 strain), doubling the median number of oocysts [22]. Silencing *TEP1 *in *An. gambiae *has a more dramatic effect (4–5 fold increase) on *P. berghei *infection [1]. Furthermore, silencing *TEP1 *in *An. gambiae *(G3 strain) does not enhance infection with *P. falciparum *(NF54 strain), indicating that there are differences in compatibility between particular strains of *An. gambiae *and *P. falciparum *(M. Povelones and A. Molina-Cruz, unpublished).

Over activation of the Rel2 pathway by silencing *Caspar*, a critical suppressor of this cascade, drastically reduces *P. falciparum *(NK54 strain) infection in *An. gambiae *(Keele strain), *An. albimanus *(Santa Tecla strain) and *An. stephensi *mosquitoes [22]. Double silencing experiments in *An. gambiae *(Keele strain) females, in which *Caspar *and *TEP1 *(or other effectors of the Rel2 pathway) were co-silenced, rescues the effect of *Caspar*, indicating that *TEP1 *is an important effector of this response. The fact that strong activation of the Rel2 pathway can very effectively prevent infection in several mosquito species that are natural vectors of *P. falciparum *[22], begs the question of why this immune response is not effective preventing disease transmission under natural field conditions.

It has been proposed that *P. falciparum *parasites have evolved specific mechanisms to modulate activation of the *An. gambiae *immune system as they adapted to their natural mosquito vector [23,24]. The observation that *P. falciparum *strains from the New World, such as the Brazilian *P. falciparum *7G8 strain, are melanized very effectively by the *An. gambiae *L35 strain but not those of African origin [9] adds support to the adaptation hypothesis. Recent experiments revealed that *LRIM1 *can also mediate immune responses against *P. falciparum*, because silencing this gene in *An. gambiae *L35 females infected with the Brazilian *P. falciparum *7G8 strain completely reverts the melanization phenotype and results in live oocysts (A. Molina-Cruz, A and C. Barillas-Mury, unpublished). Selection for refractoriness to *P. cynomolgy *resulted in a strain of *An. gambiae *that is also refractory to multiple *Plasmodium *species. *LRIM1 *also mediates the antiparasitic responses of *Anopheles quadriannulatus *to *P. berghei *infection [25]. These findings indicate that some genes, such as TEP1/*LRIM1*, are broad mediators of antiparasitic responses against several different *Plasmodium *parasites in different mosquito strains.

Under natural conditions, *P. falciparum *parasites must avoid or withstand the antiparasitic responses of *An. gambiae *to complete their life cycle and this is likely to exert selective pressure on parasite populations. For example, in Southern Mexico, three genetically distinct *P. vivax *populations have been identified, and experimental infections indicate that parasites are most compatible with sympatric mosquito species [26]. The authors propose that reciprocal selection between malaria parasites and mosquito vectors has led to local adaptation of parasites to their vectors [26]. Thus, it is likely that in well-adapted systems there is some level of immune evasion and/or suppression, and this could explain why silencing some genes involved in immunity (*LRIM1, CTL4*) or oxidative stress (*OXR1, GSTT1 and GSTT2*) in *An. gambiae *(G3) females, has little effect on *P. falciparum *(3D7 strain) infection.

There is also increasing evidence from many different studies that the interaction between *Plasmodium *parasites and the mosquito immune system it is a strong determinant of vectorial capacity. Nevertheless, the extent to which the mosquito immune system is effectively reducing *Plasmodium *infection is very variable, even between particular parasite and mosquito strains. These differences in compatibility need to be evaluated and carefully considered when selecting an experimental animal model to study malaria transmission.

## Methods

### Mosquito rearing

*An. gambiae *(G3 strain) and *An. stephensi *(Nijmegen Sda500) mosquitoes were raised at 28°C, 75% humidity under a 12-hour light/dark cycle and maintained on a 10% sucrose solution during adult stages.

### *P. berghei *and *P. yoelii yoelii *GFP 17XNL infections

Either wild-type or GFP-*P. berghei *(ANKA 2.34 strain) [27] and the GFP-*P. yoelii yoelii *17X nonlethal transgenic strain [28] were maintained by serial passage in 3- to 4-week-old female BALB/c mice or as frozen stocks. Mice parasitemias were monitored by light microscopy using air-dried blood smears that were methanol fixed and stained with 10% Giemsa. Female mosquitoes (4–5 days old) were fed on gametocytemic mice 2–3 days after blood inoculation from infected donor mice when parasitemias were between 5–10%. Mosquitoes infected with *P. berghei *or *P. yoelii *were kept at 21°C or 24°C, respectively, and midguts dissected 6–7 days post infection. Infection levels were determined by fluorescent (live oocyst) and light (melanized parasites) microscopy. The distribution of oocyst numbers in the different experimental groups was compared using the nonparametric Kolmogorov-Smirnov statistical test.

### Mosquito midgut genomic DNA extraction for quantitative real-time PCR (qPCR)

Individual midguts (without blood) were placed into microcentrifuge tubes containing 10 μl of HotSHOT alkaline lysis reagent (25 mM NaOH, 0.2 mM EDTA, pH 12.0) [29]. The tubes were boiled for 5 min and immediately placed on ice; 10 μl of HotSHOT neutralizing reagent (40 mM Tris-HCl, pH 5.0) was added to each tube. The samples were centrifuged and stored at -20°C.

### Determination of *P. berghei *infection by qPCR

For the *GSTT1 *silencing experiment, mice were infected wild-type *P. berghei *(non-GFP parasites, Anka 2.34 parasites), and the level of infection in mosquitoes was determined by qPCR 6 days post infection. Genomic DNA was obtained from infected midguts, and the abundance of *P. berghei *28S RNA relative to *An. gambiae *S7 ribosomal protein was determined. The DyNAmo SYBR Green qPCR Master mix (Finnzymes, Espoo, Finland) was used to amplify the genomic DNA, and samples were run on a MJ Research Detection system according to the manufacturer's instructions (Bio-Rad, Hercules, CA). *P. berghei *28S RNA primer sequence (5/ to 3/), Fw-GTGGCCTATCGATCCTTTA and Rev: 5/GCGTCCCAATGA TAGGAAGA). Two μl of midgut genomic DNA was used to detect the number *P. berghei *28S gene copies and 1 μl to determine the copies of *An. gambiae *ribosomal protein S7 gene in a 20-μl PCR reaction. All *P. berghei *28S values shown were then normalized relative to the number of copies of S7 in the sample. The distribution of parasite/midgut genome in control (*dsLacZ *injected) and *dsGSTT2 *silenced were compared using the Kolmogorov-Smirnov test.

### Experimental infection of *An. gambiae *mosquitoes with *P. falciparum*

*An. gambiae *(G3) female mosquitoes were infected with *P. falciparum *by feeding them gametocyte cultures using an artificial membrane feeding system. The *P. falciparum *(3D7 strain) was maintained in O+ human erythrocytes using RPMI 1640 medium supplemented with 25 mM HEPES, 50 mg/L hypoxanthine, 25 mM NaHCO_3_, and 10%(v/v) heat-inactivated type O+ human serum [30,31]. Gametocytogenesis was induced following the procedure of Ifediba and Vanderberg [32]. Mature gametocyte cultures (stages IV and V) that were 14–16 days old were used to feed mosquitoes in 37°C warmed membrane feeders for 30 minutes. To determine the level of infection, the midguts were dissected and stained with 0.05% (w/v) mercurochrome in water and oocysts counted by light microscopy 7–9 days post blood feeding. Distribution of oocyst numbers per midgut was analyzed using the Kolmogorov-Smirnov test.

### dsRNA synthesis

cDNA fragments of 500–600 bp were amplified for each gene using the primers shown in Additional File 1 and cDNA from 4-day-old *An. gambiae *females as template. The cDNA fragments were cloned into the pCR II-TOPO^® ^vector (Invitrogen, Carlsbad, CA) and T7 sites introduced at both ends using the following vector primers (5' to 3') to amplify the cDNA insert; M13-Fw: GTAAAACGACGGCCAGT and T7-M13Rev: CTCGAGTAATACGACTCACTA TAGGGCAGGAAACAGCTATGAC. dsRNA was synthesized and purified using the MEGAscript kit (Ambion, Austin, TX). The eluted dsRNA was further cleaned and concentrated to 3 μg/μl using a Microcon YM-100 filter (Millipore, Bedford, MA).

### Silencing *An. gambiae *genes

dsRNA (207 ng in 69 nl) for each of the genes tested was injected into the thorax of cold-anesthetized 1- to 2-day-old female mosquitoes using a nano-injector (Nanoject; Drummond Scientific, Broomall, PA). In each experiment, a control group was injected with *dsLacZ *or *dsGFP *to serve as reference for intensity of infection. Gene silencing was confirmed 4 days after dsRNA injection by RT-qPCR using the ribosomal S7 gene for normalization. Poly(A) mRNA was isolated from groups of 10 adult females using Oligotex-dT beads (Qiagen, Valencia, CA) following the manufacturer's instructions. First-strand cDNA was synthesized using random hexamers and Superscript II reverse transcriptase (Invitrogen). The primers used for each gene are shown in Additional File 2. Gene expression was assessed by SYBR green qPCR (DyNAmo HS; New England Biolabs, Beverly, MA) in a Chromo4 system (Bio-Rad). PCR involved an initial denaturation at 95°C for 15 minutes, 44 cycles of 10 seconds at 94°C, 20 seconds at 58°C, and 30 seconds at 72°C. Fluorescence readings were taken at 72°C after each cycle. A final extension at 72°C for 5 minutes was completed before deriving a melting curve (70°C–95°C) to confirm the identity of the PCR product. qPCR measurements were made in duplicate.

### Silencing *An. stephensi *genes

Because all the genes tested are highly conserved across species, we tested whether it was possible to silence *An. stephensi *genes by injecting them with dsRNA from orthologous genes of *An. gambiae. An. stephensi *female mosquitoes (1–2 days old) were injected with dsRNA from *An. gambiae *cDNAs following the same procedure described above. Silencing efficiency was determined using qPCR 4 days after mosquitoes were injected with dsRNA. For the initial evaluation, the same primers and conditions as for *An. gambiae *were used, except for a lower annealing temperature (52°C instead of 58°C). For *OXR1*, a strong peak was obtained using the same primers as for *An. gambiae*, but for all other genes, several primer combinations from well conserved regions had to be designed to obtain efficient amplification that generated a single band of the expected molecular weight. For *GSTT1*, in was necessary to clone a fragment of *An stephensi *cDNA using the following degenerate primers (5/ to 3/), Fwd: CTGGCGGAAAGT GTKGCCAT and Rev: GGCCGCAGCCASACGTACTGGAA. A 180-bp fragment was amplified, sequenced, and used to generate a primer combination that would efficiently amplify AsGSTT1. Sequences of all primer sets used for qRT-PCR analysis with *An. stephensi *templates are shown in Additional File 3. Silencing efficiency in *An. gambiae *and *An. stephensi*, shown in Additional File 4, ranged from 55–98% and from 56–84%, respectively.

## Abbreviations

ADP: adenosine diphosphate; *APL1*: Anopheles Plasmodium-responsive leucine-rich repeat 1; *ArgK*: arginine kinase; ATP: adenosine triphosphate; cDNA: complimentary DNA; *CTL4*: C-type lectin 4; DIC: differential interference contrast; *dsArgK, ArgK *dsRNA-injected mosquitoes; *dsCTL4*: C-type lectin 4 dsRNA-injected mosquitoes; *dsGFP, GFP *dsRNA-injected mosquitoes; *dsGSTT1*: glutathione-S-transferase theta 1 dsRNA-injected mosquitoes; *dsGSTT2*: glutathione-S-transferase theta 2 dsRNA-injected mosquitoes; *dsHsc-3*: heat-shock cognate-3dsRNA-injected mosquitoes; *dsLacZ*: β-galactosidasedsRNA-injected mosquitoes; *dsLRIM1*: leucine-rich repeat immune protein 1 dsRNA-injected mosquitoes; *dsLRIM2*: leucine-rich repeat immune protein 2 dsRNA-injected mosquitoes; *dsOXR1: oxidation resistance 1 *dsRNA-injected mosquitoes; dsRNA: double-stranded RNA; *dsSol.trsp*: solute transporter dsRNA-injected mosquitoes; *dsTEP1*: thioester-containing protein *1 *dsRNA-injected mosquitoes; *dsTetrasp*: tetraspanin dsRNA-injected mosquitoes; GFP-*P. yoelii yoelii *17XNL: *Plasmodium yoelii yoelii *17X nonlethal transgenic strain constitutively expressing green fluorescent protein; *GSTT*: gene family, glutathione-S-transferase of the theta class gene family; *GSTT1*: glutathione-S-transferase theta 1; *GSTT2*: glutathione-S-transferase theta 2; *Hsc-3*: heat-shock cognate-3; KS: Kolmogorov-Smirnov; *LRIM1*: leucine-rich repeat immune protein 1; *LRIM2*: leucine-rich repeat immune protein 2; mRNA: messenger RNA; *OXR1*: oxidation resistance 1; PCR: polymerase chain reaction; qPCR: quantitative real-time PCR; qRT-PCR: quantitative real-time reverse-transcriptase PCR; ROS: reactive oxygen species; RPMI: Royal Park Memorial Institute; S7, protein from the small ribosomal subunit S7; *Sol.trsp*: solute transporter; *TEP1*: thioester-containing protein 1; *Tetrasp*: tetraspanin; TM: transmembrane domain.

## Authors' contributions

GJ-G carried out most of the experimental work, data analysis, and drafted the manuscript. JR performed most of the experiments involving silencing of *GSTT1 *and helped with midgut dissections and oocyst counting. GN and GJ-G performed the *P. yoelii *infections in *An. gambiae *and *An. stephensi*. MP and GJ-G silenced *TEP1*, *LRIM1*, and *LRIM2 *in *P. yoelii*-infected *An. gambiae*. A M-C prepared the *P. falciparum *gametocyte cultures. C B-M contributed with experimental design, data analysis, image processing, assembly of final figures, and writing the manuscript.

## Supplementary Material

Additional file 1 **Validation of gene silencing in *An. gambiae *and *An. stephensi***. The data indicate the silencing efficiency of several genes after dsRNA injection in *An. gambiae *and *An. stephensi*, relative to a control group injected with dsLacZ. Click here for file

Additional file 2 **Primers used to generate dsRNA using *An. gambiae *cDNA as template**. The data indicate the sequence of the primers used to generate dsRNA using *An. gambiae *cDNA as template. Click here for file

Additional file 3 **Primers used to determine gene expression by qRT-PCR and validate gene silencing in *An. gambiae***. The data indicate the sequence of the primers used for gene expression analysis by qRT-PCR to validate gene silencing in *An. gambiae*. Click here for file

Additional file 4 **Primers used to determine gene expression by qRT-PCR and validate gene silencing in *An. stephensi***. The data indicate the sequence of the primers used for gene expression analysis by qRT-PCR to validate gene silencing in *An. stephensi*. Click here for file
