# Supplementary material for: Mosquito immune responses and compatibility between Plasmodium parasites and anopheline mosquitoes
Source: BMC Microbiol. 2009 Jul 30;9:154. doi: 10.1186/1471-2180-9-154 (PMC2782267; doi:10.1186/1471-2180-9-154)
Supplement: Additional file 1 — Validation of gene silencing in An. gambiae and An. stephensi. The data indicate the silencing efficiency of several genes after dsRNA injection in An. gambiae and An. stephensi, relative to a control group injected with dsLacZ. [file 1471-2180-9-154-S1.pdf]

Additional file 1

Validation of gene silencing in *An. gambiae* and *An. stephensi*. Silencing efficiency is expressed as the percent reduction in whole body mRNA 4-5 days after injection of dsRNA, relative to a control group injected with dsLacZ.

Gene	% silencing *An. gambiae*	% silencing *An. stephensi*
OXR1	75%	56%
Hsc-3	98%	75%
GSTT1	60%	75%
GSTT2	55%	60%
LRIM1	86%	84%
CTL4	70%	60%
