# Supplementary material for: Mosquito immune responses and compatibility between Plasmodium parasites and anopheline mosquitoes
Source: BMC Microbiol. 2009 Jul 30;9:154. doi: 10.1186/1471-2180-9-154 (PMC2782267; doi:10.1186/1471-2180-9-154)
Supplement: Additional file 2 — Primers used to generate dsRNA using An. gambiae cDNA as template. The data indicate the sequence of the primers used to generate dsRNA using An. gambiae cDNA as template. [file 1471-2180-9-154-S2.pdf]

Additional file 2

Primers used to generate dsRNA using *An. gambiae* cDNA as template.

Predicted protein	AGAP ID*	Primer sequence
OXR1	AGAP001751	Fw: 5’ AAAGCGACCCTGTTTGCGTC 3’<br>Rv: 5’ ACGAGCGTTTTGATGACGAAATC 3’
Arginine kinase	AGAP005627	Fw: 5’ GCTCTGAAGAACAAGAAGACCTC 3’<br>Rv: 5’ GGCCCTCCTTGAACAGGAAGTG 3’
Solute Transporter	AGAP010892	Fw: 5’ CGGCTCGGGCGTAAGTTTTTC 3’<br>Rv: 5’ CGGTATCGGTGGTGTGAAGTTG 3’
Tetraspanin	AGAP005233	Fw: 5’ CCACCAGTGGTACTGATTGTCGTTG 3’<br>Rv: 5’ GTGGAAATGGTCCTCTAGCTTCATC 3’
Hsc-3	AGAP004192	Fw: 5’ AAGAAGAAGAAGGGCAAGGACATC 3’<br>Rv: 5’ GATCAGCTTGGTCATCACTCCG 3’
GSTT1	AGAP000761	Fw: 5’ TGGGCAAAGGAGAGCATCTGAC 3’<br>Rv: 5’ GCCTGGTCGTAGTATGGATTGG 3’
GSTT2	AGAP000888	Fw: 5' CAGCACAAGACGGACGAATAC 3'<br>Rv: 5' TAATGATTTCGCCTGCTCAATCTCGC 3'
LRIM1	AGAP006348	Fw: 5’ AATATCTATCTCGCGAACAATAA 3’<br>Rv: 5’ TTGGCACGGTACACTCTTCCT 3’
CTL4	AGAP005335	Fw:5' GTTAGCAGCATTGGGATTACCCTCG 3'<br>Rv: 5’ GAAGTCGCAACCCAGCTCA TTGTAG 3’
TEP1	AGAP010815-PA	Fw 5'-TTTGTGGGCCTTAAAGCGCTG-3'<br>Rv 5'-ACCACGTAACCGCTCGGTAAG-3'
APL1/LRIM2	AGAP007033	Fw 5'-GCTTACGCGCACACTATTCA-3'<br>Rv-5'-GCTATTGTGCGATGCGTCTA-3'
