# Supplementary material for: Mosquito immune responses and compatibility between Plasmodium parasites and anopheline mosquitoes
Source: BMC Microbiol. 2009 Jul 30;9:154. doi: 10.1186/1471-2180-9-154 (PMC2782267; doi:10.1186/1471-2180-9-154)
Supplement: Additional file 3 — Primers used to determine gene expression by qRT-PCR and validate gene silencing in An. gambiae. The data indicate the sequence of the primers used for gene expression analysis by qRT-PCR to validate gene silencing in An. gambiae. [file 1471-2180-9-154-S3.pdf]

Additional file 3

Primers used to determine gene expression by qRT-PCR and validate gene silencing in *An. gambiae.*

Predicted protein	Gene ID	Primer sequence
OXR1	AGAP001751	Fw: 5' GCACAGATGACTACAGAAAAGCGAC 3'<br>Rv: 5' AACACGAGAGACCACGAGTATCCC 3'
Arginine kinase	AGAP005627	Fw: 5' TCGACGATCACTTCCTGTTCAAG 3'<br>Rv: 5' GTTGTCGTTGTGGTAGATACCGC 3'
Solute Transporter	AGAP010892	Fw: 5' TTCGCTTCCTGAACGGTGTG 3'<br>Rv: 5' TGAACGACGGTATGGAGCAGAC 3'
Tetraspanin	AGAP005233	Fw: 5' TGATGAAGCTAGAGGACCATTTCC 3'<br>Rv: 5' CTTTTGATGGAAGATGACAGCCAG 3'
Hsc-3	AGAP004192	Fw: 5' AAGAAGAAGAAGGGCAAGGACATC 3'<br>Rv: 5' GATCAGCTTGGTCATCACTCCG 3'
GSTT1	AGAP000761	Fw: 5' AAACCTAACCCAGTGGATGG 3'<br>Rv: 5' TAGCTTTGCTAGCCGTGTCC 3'
GSTT2	AGAP000888	Fw: 5' GTCCAGCAACTTGAACTTGAAACC 3'<br>Rv: 5' AGGGGATCTTGTTGGTCGATAAG 3'
LRIM1	AGAP006348	Fw: 5' CATCCGCGATTGGGATATGT 3'<br>Rv: 5' CTTCTTGAGCCGTGCATTTTC 3'
CTL4	AGAP005335	Fw: 5' CGATACGAGAGCGGCAGTAT 3'<br>Rv: 5' TCTGCATCGAAACTGGGTAA 3'
