# Supplementary material for: Mosquito immune responses and compatibility between Plasmodium parasites and anopheline mosquitoes
Source: BMC Microbiol. 2009 Jul 30;9:154. doi: 10.1186/1471-2180-9-154 (PMC2782267; doi:10.1186/1471-2180-9-154)
Supplement: Additional file 4 — Primers used to determine gene expression by qRT-PCR and validate gene silencing in An. stephensi. The data indicate the sequence of the primers used for gene expression analysis by qRT-PCR to validate gene silencing in An. stephensi. [file 1471-2180-9-154-S4.pdf]

Additional file 4

Primers used to determine gene expression by qRT-PCR and validate gene silencing in *An. stephensi.*

Gene	Primer sequence
OXR1	Fw: 5’ GCACAGATGACTACAGAAAAGCGAC 3’<br>Rv: 5’ AACACGAGAGACCACGAGTATCCC 3’
Hsc-3	Fw: 5’ GTGTACAAGAACGGGCGCGTG 3’<br>Rv: 5’ CACGCGCCCGTTCTTGTACAC 3’
GSTT1	Fw: 5’ TATCTGTGCCGGGAGTATAC 3’<br>Rv: 5’ GGCCGCAGCCACACGTACTGGAA 3’
GSTT2	Fw: 5’ AATAGCCGGGTACGATCCGTG 3’<br>Rv: 5’ AACGTATTTGTGCGCCTCGTCG 3’
LRIM1	Fw: 5’ CATCCGCGATTGGGATATGT 3’<br>Rv: 5’ CTTCTTGAGCCGTGCATTTTC 3’
CTL4	Fw: 5’ ATGCAGATCTCAAACATATTTG 3’<br>Rv: 5’ GTTTTGGGTAATCATTTCG 3’
TEP1	Fw 5'-AAAGCTGTTGCGTCAGGG-3'<br>Rv 5'-TTCTCCCACACACCAAACGAA-3'
APLI/LRIM2	Fw 5'-GCAAAGAAAGTGACAAGCCGTAT-3'<br>Rv 5'-CGCTCGTCAGGGCAATGTA-3'
